# Spatial-Channel Multiscale Transformer Network for Hyperspectral Unmixing

**DOI:** 10.3390/s25144493

**Published:** 2025-07-19

**Authors:** Haixin Sun, Qiuguang Cao, Fanlei Meng, Jingwen Xu, Mengdi Cheng

**Affiliations:** College of Electronic and Information Engineering, Changchun University, Changchun 130022, China; 230401165@mails.ccu.edu.cn (Q.C.); mengfl@ccu.edu.cn (F.M.); 230401168@mails.ccu.edu.cn (J.X.); 230402194@mails.ccu.edu.cn (M.C.)

**Keywords:** global contextual information, multiscale transformer, hyperspectral unmixing, spatial–spectral modeling, multi-head self-attention

## Abstract

In recent years, deep learning (DL) has been demonstrated remarkable capabilities in hyperspectral unmixing (HU) due to its powerful feature representation ability. Convolutional neural networks (CNNs) are effective in capturing local spatial information, but limited in modeling long-range dependencies. In contrast, transformer architectures extract global contextual features via multi-head self-attention (MHSA) mechanisms. However, most existing transformer-based HU methods focus only on spatial or spectral modeling at a single scale, lacking a unified mechanism to jointly explore spatial and channel-wise dependencies. This limitation is particularly critical for multiscale contextual representation in complex scenes. To address these issues, this article proposes a novel Spatial-Channel Multiscale Transformer Network (SCMT-Net) for HU. Specifically, a compact feature projection (CFP) module is first used to extract shallow discriminative features. Then, a spatial multiscale transformer (SMT) and a channel multiscale transformer (CMT) are sequentially applied to model contextual relations across spatial dimensions and long-range dependencies among spectral channels. In addition, a multiscale multi-head self-attention (MMSA) module is designed to extract rich multiscale global contextual and channel information, enabling a balance between accuracy and efficiency. An efficient feed-forward network (E-FFN) is further introduced to enhance inter-channel information flow and fusion. Experiments conducted on three real hyperspectral datasets (Samson, Jasper and Apex) and one synthetic dataset showed that SCMT-Net consistently outperformed existing approaches in both abundance estimation and endmember extraction, demonstrating superior accuracy and robustness.

## 1. Introduction

Hyperspectral images (HSIs) are three-dimensional data cubes that contain both spatial and spectral information, typically consisting of tens to hundreds of spectral bands. These bands typically span a spectral range from the visible to the short-wave infrared regions, approximately from 400 to 2500 nm [1]. Unlike traditional RGB images that are limited to three channels (red, green, and blue), HSIs provide detailed spectral signatures of materials along with their spatial distribution, making them widely applicable in diverse fields such as food safety [2], environmental monitoring [3], and mineral exploration [4]. However, due to limitations in imaging technology, HSIs generally suffer from a low spatial resolution [5], where each pixel often contains a mixture of spectral information from multiple materials—commonly referred to as mixed pixels [6]. The presence of a large number of mixed pixels significantly degrades the performance of HSI-based applications. Therefore, it is essential to decompose these mixed pixels to retrieve the pure spectral components (known as endmembers) and their corresponding proportions within each pixel, a process known as hyperspectral unmixing (HU). The task of extracting the pure spectral signatures from mixed pixels is referred to as endmember extraction [7], while estimating their proportion in each pixel is called abundance estimation [8]. Under physically meaningful constraints, abundance values are typically required to satisfy two conditions: the Abundance Nonnegative Constraint (ANC) and the Abundance Sum-to-one Constraint (ASC) [9,10].

In HU tasks, the linear mixing model (LMM) [11] has become the most widely adopted unmixing framework due to its clear physical interpretability and computational simplicity. Based on the LMM assumption, numerous unmixing approaches have been proposed to effectively estimate endmember spectra and their corresponding abundance distributions.

Traditional HU methods include geometric approaches, statistical models [12], and sparse regression-based techniques [13,14]. Among geometric methods, vertex component analysis (VCA) [15] and fully constrained least squares unmixing (FCLSU) [16] are widely used. VCA projects the HSI onto directions orthogonal to the subspace formed by the selected endmembers and iteratively extracts potential endmember spectra. Under the assumption that pure pixels exist, this method can effectively identify pure material spectra in the scene, providing a basis for subsequent abundance estimation. FCLSU, on the other hand, performs least-squares regression to estimate abundances given known endmembers, while enforcing the non-negativity and sum-to-one constraints. However, in practical scenarios, pixels composed entirely of a single material are rarely observed, making the pure-pixel assumption often invalid and limiting the applicability of VCA. In addition, the performance of FCLSU heavily relies on the accuracy of the extracted endmembers. If the estimated endmembers deviate from the actual spectra, the resulting abundance maps may also suffer in accuracy, thereby degrading the overall unmixing performance. To address these limitations, a family of methods based on non-negative matrix factorization (NMF) has been proposed [17,18,19,20]. Unlike geometric approaches, NMF does not depend on the pure-pixel assumption. Instead, it decomposes the observed HSI into a product of two nonnegative matrices representing the endmember spectra and their abundances, respectively. This allows for a fully unsupervised estimation of both components, making NMF more robust in highly mixed scenes. Qian et al. [21] introduced the $L_{1/2}$ sparsity constraint into NMF for HU, referred to as $L_{1/2}$-NMF, which improves the unmixing accuracy by promoting sparsity in abundance estimation. Compared with the traditional $L_{1}$-norm, the $L_{1/2}$-norm induces stronger sparsity and is mathematically non-convex. Rajabi and Ghassemian [22] proposed a multilayer extension called Multilayer NMF (MLNMF), which iteratively factorizes the observation matrix into multiple hierarchical layers to refine unmixing performance. Sparse regression-based methods assume that each pixel can be represented as a linear combination of a small subset of endmembers from a predefined spectral library. These methods aim to identify both the contributing endmembers and their corresponding proportions through sparse optimization. Bioucas-Dias and Figueiredo [23] proposed Sparse Unmixing via Variable Splitting and Augmented Lagrangian (SUnSAL), which incorporates a $L_{1}$-norm regularization term to enforce sparsity. SUnSAL is particularly effective when a large spectral library is available and pure pixels are difficult to obtain.

Recently, deep learning (DL) networks have provided effective solutions for HU [24,25]. A typical DL-based unmixing framework adopts an autoencoder (AE) architecture, which consists of an encoder and a decoder. The encoder is responsible for extracting low-dimensional representations from the input HSI, which correspond to abundance estimations. The decoder reconstructs the original HSI using the estimated abundances and the learned endmember spectra [26]. Based on the AE framework, the integration of different feature extraction modules and the design of tailored loss functions can further improve unmixing performance [27,28]. For instance, Qu and Qi [29] proposed an untied denoising autoencoder with sparsity (uDAS), which introduces an $L_{21}$-norm constraint to enhance the accuracy of abundance estimation. This regularization helps reduce redundancy in the learned features and improves the robustness and precision of the encoder in estimating abundance maps. Su et al. [30] introduced the Stacked Nonnegative Sparse Autoencoders (SNSAEs), which employ an end-to-end fully connected (FC) AE structure. Without explicitly incorporating spatial modeling, this approach leverages spectral feature learning to effectively estimate abundance representations under unsupervised conditions, achieving robust unmixing performance for HSIs.

Early AE-based unmixing methods primarily relied on FC layers to construct the encoder and decoder. During the processing of HSIs, each pixel (or spectral vector) is often treated as an independent sample, or the entire HSI is flattened into a long vector for spectral feature learning. However, these approaches typically ignore the spatial relationships between neighboring pixels. To more effectively leverage the valuable spatial information in HSIs, researchers have introduced convolutional neural networks (CNNs) into AE architectures to further improve HU performance. Palsson et al. [31] proposed a CNN Autoencoder Unmixing (CNNAEU) framework, which integrates convolutional encoders and decoders to extract spatial features and reconstruct spectral information. This approach enables a more accurate abundance estimation by jointly learning spatial–spectral representations. Rasti et al. [32] introduced an unsupervised HU method based on deep CNNs, termed Unmixing Deep Prior (UnDIP). By exploiting the structural prior embedded in the network itself, UnDIP models the relationships between endmembers and abundances without external supervision, thereby enhancing unmixing accuracy and robustness. Gao et al. [33] proposed a Cycle-Consistency Unmixing Network (CyCU-Net), which cascades two autoencoders for HU and introduces cycle-consistency constraints through spectral and abundance reconstruction losses. This framework strengthens the representational capacity of both endmembers and abundances, improving both the accuracy and stability of unmixing. While CNN-based AE unmixing methods are capable of extracting local spatial features, such feature extraction is primarily dependent on the size of convolutional kernels, which inherently rely on limited receptive fields. This constraint hampers their ability to capture long-range spatial dependencies and global spectral relationships, leading to the loss of critical contextual features during unmixing. Moreover, due to the high dimensionality of HSIs, although some CNN-based methods enhance global modeling via encoder–decoder or residual structures, they still rely on stacked local operations, whereas the transformer captures long-range spatial–spectral dependencies more efficiently through self-attention.

Transformer architectures have rapidly gained attention in remote sensing image processing due to their superior ability to model long-range dependencies and capture global contextual features. In recent years, several studies have explored the application of transformers to HU and have achieved promising results [34,35,36]. Ghosh et al. [37] proposed the first hybrid HU model that combines transformer and CNN architectures. In this approach, the multi-head self-attention mechanism of the transformer is employed to complement the limited receptive field of the CNN, thereby enhancing the robustness and accuracy of the unmixing process. This work laid a foundation for subsequent transformer-based HU research. Recently, there has been increasing interest in integrating CNNs and transformers to further improve unmixing performance. Hu et al. [34] introduced the Multiscale Convolution Attention Network (HUMSCAN), which consists of an endmember estimation sub-network and an abundance estimation sub-network. By leveraging multiscale convolutions to extract spatial features at different scales and attention mechanisms to enhance salient feature representations, HUMSCAN effectively improves HU performance. Yang et al. [35] proposed the Cascaded Dual-Constrained Transformer Autoencoder (CDCTA), which constructs a progressive, cascaded structure by stacking multiple transformer encoder–decoder modules. This design enhances the model’s depth and expressive capacity for complex mixed pixels. Moreover, CDCTA incorporates two additional constraints—endmember separability and abundance sparsity—into the network to improve the accuracy of both endmember extraction and abundance estimation. Wang et al. [38] proposed the Multiscale Aggregation Transformer Network (MAT-Net), which fully exploits CNN-extracted spectral and multiscale spatial features and then fuses them using a transformer encoder. MAT-Net features a dual-stream, multi-branch CNN encoder and an enhanced multiscale self-attention module that adaptively aggregates information across scales, achieving effective and accurate endmember extraction and abundance estimation. Gan et al. [39] proposed a Channel Multi-Scale Dual-Stream Autoencoder (CMSDAE), which performs multiscale feature modeling along the channel dimension to effectively reduce redundancy in the spatial domain and enhance feature representation, thereby improving the accuracy of endmember extraction and abundance estimation. Hadi et al. [40] introduced a Dual-branch Spectral–Spatial Feature Fusion Transformer (DSSFT), which integrates spectral and spatial information through two parallel branches. The spectral branch employs a self-attention mechanism to model complex spectral variations and enhance endmember identification, while the spatial branch adopts patch-level embedding to capture global spatial context, improving the discriminative ability for endmembers and abundances in heterogeneous regions. In addition, Xiang et al. [41] proposed an Endmember-Oriented Transformer Network (EOT-Net), which combines endmember bundle modeling with directional subspace projection to extract endmember-specific features and incorporates a low-redundancy attention mechanism to enhance feature discrimination, effectively improving unmixing accuracy.

However, existing HU methods that combine CNNs and transformers often fail to fully exploit the channel-wise information of HSIs, and they lack dynamic interaction mechanisms for multiscale global contextual modeling. These limitations restrict the joint representation capability of spatial and spectral features in HSIs. To address this issue, we propose a Spatial-Channel Multiscale Transformer Network (SCMT-Net) for HU. Specifically, a spatial multiscale transformer (SMT) module is first introduced to learn spatial features of the HSI, followed by a channel multiscale transformer (CMT) module designed to capture long-range dependencies across spectral channels. The integration of these two modules enables global and dynamic modeling across spatial and spectral dimensions. Moreover, a multiscale multihead self-attention (MMSA) mechanism is incorporated into both the SMT and CMT modules to effectively extract rich spatial–spectral contextual information. Finally, an efficient feed-forward network (E-FFN) is employed to enhance inter-channel information flow and feature fusion, thereby further improving unmixing performance.

The main contributions of this article are summarized as follows:

1. We propose a novel unmixing network, SCMT-Net, which integrates a CFP module and a spatial-channel multiscale transformer module to enable the collaborative modeling of local details and a global context, achieving the dynamic learning of multiscale spatial and spectral relationships.

2. A CMT module is designed to deeply capture long-range dependencies across HSI spectral channels. By combining it with the SMT module, we construct the core SCMT module, which significantly enhances the modeling capacity of spatial-channel global relationships in complex scenarios.

3. A new MMSA module is introduced, embedding multiscale global contextual and channel information into the attention mechanism to capture rich spatial–spectral features. Additionally, an E-FFN is incorporated to further strengthen inter-channel information interaction, thereby improving overall unmixing performance.

The remainder of this article is organized as follows. Section 2 introduces the background and related concepts of HU. Section 3 presents the architecture and fundamental principles of the proposed SCMT-Net. Section 4 discusses the experimental results on three real-world hyperspectral datasets and one synthetic dataset, including comparisons with several representative HU methods and ablation studies on SCMT-Net. Finally, Section 5 concludes the article with a summary of key findings.

## 2. Background

In HSIs, due to the limited spatial resolution and the mixed distribution of surface materials, each pixel typically contains a mixture of multiple pure spectral components (endmembers). The most commonly used LMM assumes that the observed pixel spectrum can be represented as a weighted linear combination of several endmember spectra. Its mathematical expression is given by

(1) Y=EA+N

The input HSI is denoted as $X\in R^{L\times H\times W}$, where *H*, *W*, and *L* represent the height, width, and number of spectral bands of the original HSI, respectively. The HSI can be mathematically reshaped into a matrix $Y\in R^{L\times n}$, where n=H·W denotes the total number of pixels and *L* represents the number of spectral bands. It is important to note that this reshaping is used solely for notational purposes; in practice, the encoder retains the spatial structure before explicitly flattening the input for the transformer. The endmember matrix is denoted as $E\in R^{L\times R}$, where *R* represents the number of endmembers present in the HSI. The corresponding abundance cube (i.e., the stack of R abundance maps) is represented as $M\in R^{R\times H\times W}$, which can be reshaped into a matrix $A\in R^{R\times n}$; $N\in R^{L\times n}$ represents the additive noise present in Y.

In addition, HU tasks typically require the following three physical constraints to be satisfied:

First, the endmember matrix must be non-negative, that is, E≥0; second, the abundance matrix is subject to the ANC, i.e., A≥0; finally, the ASC must also be satisfied: $1_{R}^{T}A=1_{n}^{T}$, where $1_{n}$ denotes an all-ones column vector of dimension *n*.

Although the LMM offers good physical interpretability and modeling simplicity, under non-ideal imaging conditions such as illumination variations, terrain undulations, material inhomogeneity, or multipath scattering, the actual mixing process often exhibits pixel-wise spectral variability. This leads to the inability of the LMM to accurately model such complex scenarios. To address this issue, researchers have proposed a generalized version of the LMM, which enhances the adaptability and representational capacity of the model while preserving its linear structure.

GLMM introduces scaling factors for endmembers at the pixel level, allowing endmember spectra to vary across different pixels, thereby enhancing the ability to model spectral variability in real-world scenarios. Its mathematical expression is as follows:

(2) $$x_{n}=M_{n}\cdot a_{n}+e_{n}$$

Specifically, $x_{n}\in R^{L}$ denotes the observed spectrum of the nth pixel, $M_{n}\in R^{L\times R}$ denotes the endmember spectral matrix of that pixel, $a_{n}\in R^{R}$ denotes the corresponding abundance vector, and $e_{n}$ denotes the additive noise. GLMM extends the standard LMM by introducing pixel-level endmember scaling factors, allowing endmembers to vary across different pixels, thereby enhancing the ability to represent spectral variability while preserving the linear mixing structure.

In this study, although SCMT-Net adopts the LMM as a physical foundation and constraint framework for task modeling, the network itself is essentially a nonlinear unmixing method. Its architecture integrates multiscale attention mechanisms, nonlinear activation functions, and multiscale depthwise separable convolution modules, enabling the end-to-end learning of complex nonlinear mappings from input hyperspectral images to abundance maps and endmember spectra. Therefore, SCMT-Net does not rely on the strict linear assumptions of LMM; instead, it builds upon this physical modeling basis to achieve a more expressive and flexible nonlinear modeling process. This design allows the model to maintain robust performance and generalization capability, even under complex mixing scenarios involving pixel-level endmember variability or nonlinear interactions.

## 3. Methods

**Figure 1:**
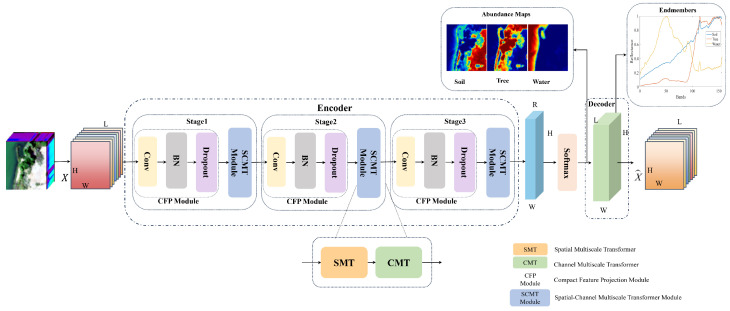
Architecture of SCMT-Net. In the abundance maps, red indicates higher abundance values while blue indicates lower abundance values.

The overall architecture of SCMT-Net is illustrated in Figure 1. SCMT-Net adopts an AE structure consisting of an encoder and a decoder. Within the encoder, the input X is first processed by the CFP module, which performs channel dimensionality reduction to extract discriminative features $X_{\mathrm{CFP}}\in R^{C\times H\times W}$, where *C* denotes the reduced number of channels. Subsequently, $X_{\mathrm{CFP}}$ is fed into the SCMT module, which sequentially incorporates the SMT and CMT modules to extract global spatial features and long-range dependencies among channels at multiple scales. This process provides enriched spatial interactions and inter-channel correlations for the unmixing task. The encoder comprises three stages, each employing depthwise separable atrous convolutions with different atrous rates to effectively capture multiscale spatial–spectral information. To satisfy the ASC and the ANC, the output of the encoder is projected back to the original HSI spatial dimensions through a convolutional layer, followed by a softmax activation to generate the final estimated abundance maps. Finally, the decoder increases the number of output channels to match the spectral dimensionality of the original HSI through a convolutional layer and simultaneously extracts the estimated endmember signatures. The following section provides a detailed discussion of the components of SCMT-Net and analyzes its key modules.

### 3.1. CFP Module

The CFP module consists of a convolutional (Conv) layer, a batch normalization (BN) layer, and a dropout layer. Specifically, the convolutional layer employs a 1 × 1 two-dimensional convolution to reduce the spectral dimensionality of the input HSI and extract essential spatial features. This is followed by batch normalization to improve training stability and mitigate gradient vanishing, and a dropout layer to reduce the risk of overfitting. The CFP module ultimately outputs low-dimensional features denoted as $X_{\mathrm{CFP}}$.

### 3.2. SCMT Module

The SCMT module is composed of two key components: SMT and CMT. This module is designed to fully exploit global feature dependencies within the HSI, thereby enhancing the feature representation capability during the HU process. The following subsections detail the fundamental procedures of the SMT and CMT modules, respectively.

#### 3.2.1. SMT Module

**Figure 2:**
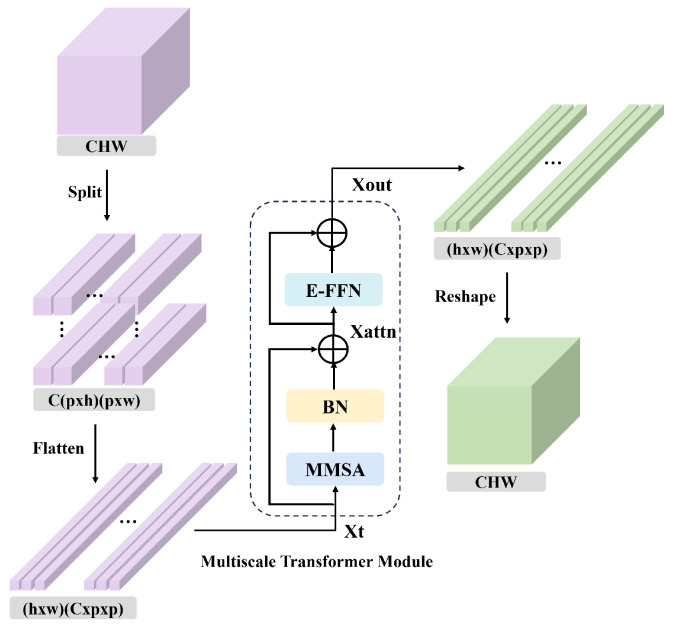
The framework of SMT.

The structure of the SMT module is illustrated in Figure 2. First, the feature map $X_{\mathrm{CFP}}$ generated by the CFP module is divided into *N* patches of size p×p, where each patch has a dimensionality of $X_{p}\in R^{C\times p\times p}$, and a total of $N=\frac{HW}{p^{2}}$ patches are obtained. All patches are flattened to form a token sequence $X_{t}\in R^{N\times (p\times p\times C)}$. This sequence is then fed into the multiscale transformer (MT) module to achieve global spatial feature modeling across all patches, thereby enhancing the contextual representation of spatial features. The MT module consists of the MMSA module, a BN layer, and the E-FFN module. Specifically, the token sequence $X_{t}$ is first processed by the MMSA module, followed by normalization and a residual connection with the original input, resulting in $X_{\mathrm{attn}}$. This intermediate output is then passed through the E-FFN module and another residual connection to obtain the final output $X_{\mathrm{out}}$. The above process can be formulated as follows:

(3) $$X_{\mathrm{attn}}=X_{t}+\mathrm{BN}\left(\mathrm{MMSA}\left(X_{t}\right)\right)$$

(4) $$X_{\mathrm{out}}=X_{\mathrm{attn}}+\left(E-\mathrm{FFN}(X_{\mathrm{attn}})\right)$$

After passing through the MT module, the feature map is reshaped back to the original spatial dimensions, thereby restoring the structural layout of the image.

(1) MMSA Module

**Figure 3:**
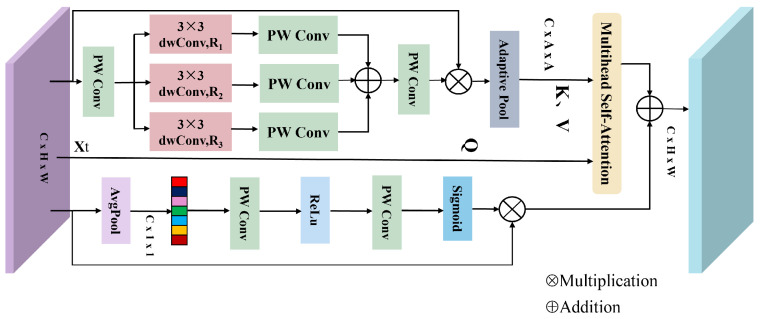
Illustration of MMSA module.

The MMSA module adopts a dual-branch architecture to extract multiscale global contextual and channel information, as illustrated in Figure 3. The upper branch is designed to capture multiscale global contextual information. Specifically, the input feature map $X_{t}$ is first processed by a pointwise convolution (PW Conv) to reduce its channel dimension to *C*/4. The resulting feature is then passed through three parallel depthwise separable atrous convolutions, each with a kernel size of 3 × 3. The atrous rates for the three convolutional branches are denoted as $R_{i}\;\left(i=\left\{1,2,3\right\}\right)$, with values {(1,3,5),(3,5,7),(5,7,9)}, corresponding to the MMSA modules integrated into each stage of the MT module.

Subsequently, a PW Conv is employed to restore the feature map to the original number of input channels, followed by an element-wise summation. The result is then processed through another PW Conv and a residual connection to obtain the final output $X_{\mathrm{Multi}}$:

(5) $$X_{i}={\mathrm{dwConv}}_{3\times 3}^{R_{i}}\left(\mathrm{PWConv}(X_{t})\right)$$

(6) $${\tilde{X}}_{i}=\mathrm{PWConv}\left(X_{i}\right)$$

(7) $$X_{\mathrm{Multi}}=\mathrm{PWConv}\left(\sum_{i=1}^{3}{\tilde{X}}_{i}\right)⊗X_{t}$$

where the symbol ⊗ denotes element-wise multiplication. $X_{i}\;\left(i=1,2,3\right)$ represent the outputs of three depthwise separable atrous convolution branches, ${\mathrm{dwConv}}_{3\times 3}^{R_{i}}\left(\cdot \right)$ denotes a 3 × 3 depthwise separable atrous convolution with an atrous rate of $R_{i}$ and ${\tilde{X}}_{i}\;\left(i\in \left\{1,2,3\right\}\right)$ denote the corresponding outputs from three PW Conv branches. $X_{\mathrm{Multi}}$ indicates the final fused feature. Subsequently, the fused feature is fed into an adaptive average pooling layer to extract multiscale features $X_{\mathrm{Ada}}\in R^{C\times (A\cdot A)}$:

(8) $$X_{\mathrm{Ada}}=\mathrm{AdaptivePool}\left(X_{\mathrm{Multi}}\right)$$

For simplicity, the flattening operation is omitted. The resulting feature $X_{\mathrm{Ada}}$ exhibits a lower spatial resolution compared to the original input $X_{t}$, where *A* is set to 9, taking the Samson dataset as an example. Note that A is empirically set as a fixed hyperparameter for each dataset, as summarized in Table 1. This feature representation captures rich multiscale contextual information derived from the input.

The matrix $X_{\mathrm{Ada}}$ is used to compute the key (K) and value (V) for the multi-head self-attention mechanism, while $X_{t}$ is used to generate the queries (Q). The computation process is formulated as follows:

(9) $$\left(Q,\;K,\;V\right)=\left(X_{t}W_{q},\;X_{\mathrm{Ada}}W_{k},\;X_{\mathrm{Ada}}W_{v}\right)$$

where $W_{q},\;W_{k},\;W_{v}$ denote the learnable weight matrices for the linear transformations. The K and V matrices incorporate multiscale contextual information, enhancing the capability of modeling global contextual features and thereby improving unmixing performance. Subsequently, the Q, K, and V matrices are fed into the multi-head self-attention module to compute the self-attention features:

(10) $${\mathrm{Attention}}_{M}=\mathrm{Softmax}\left(\frac{Q\cdot K^{T}}{\sqrt{d_{k}}}\right)\cdot V$$

where $d_{k}$ denotes the channel dimension of K, and the division by $\sqrt{d_{k}}$ can be regarded as an approximate normalization. The softmax function is applied row-wise across the matrix. For simplicity, the concept of multi-head attention is omitted in Equation (9), as discussed in [42,43]. Since the lengths of K and V are shorter than that of the input $X_{t}$, the proposed MMSA module introduces lower computational overhead compared with conventional multi-head self-attention mechanisms. Furthermore, as K and V encode rich multiscale contextual information, the proposed MMSA module is more effective in modeling global contextual dependencies, which benefits the HU task.

Inspired by SENet [44], a channel attention branch is constructed in the lower branch to efficiently capture inter-channel dependencies. The input feature $X_{t}$ is first passed through a global average pooling layer to generate a channel attention map $X_{\mathrm{Avg}}\in R^{C\times 1\times 1}$. This map is then fed into a PW Conv for channel reduction, followed by a ReLU activation and another PW Conv to restore the channel dimension to *C*. Finally, a Sigmoid activation function is applied to obtain channel-wise attention weights, which are multiplied element-wise with the original feature $X_{t}$ to produce the channel-enhanced feature map. The entire process can be described as follows.

(11) $$X_{\mathrm{Avg}}=\mathrm{Avgpool}\left(X_{t}\right)$$

(12) $$X_{C}=\mathrm{ReLU}\left(\mathrm{PWConv}\left(X_{\mathrm{Avg}}\right)\right)$$

(13) $${\mathrm{Attention}}_{C}=\mathrm{Sigmoid}\left(\mathrm{PWConv}\left(X_{C}\right)\right)⊗X_{t}$$

Finally, the features from the upper and lower branches are summed to obtain the final output of the MMSA module, denoted as $X_{\mathrm{MMSA}}$:

(14) $$X_{\mathrm{MMSA}}={\mathrm{Attention}}_{M}+{\mathrm{Attention}}_{C}$$

(2) E-FFN Module

**Figure 4:**
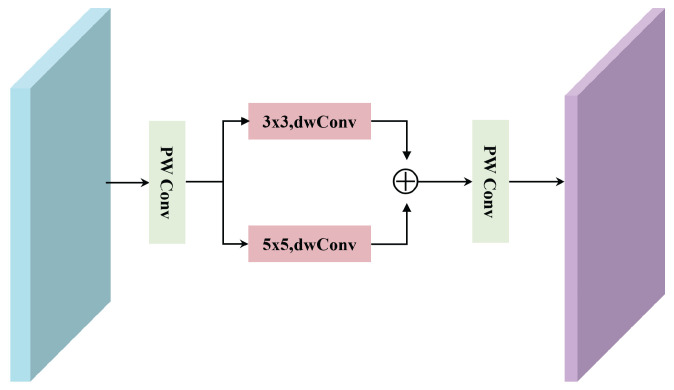
The framework of efficient feed-forward network (E-FFN).

Conventional transformers typically rely on FC layers as the FFN [40] and depend entirely on the attention mechanism to capture dependencies among pixels [41]. Although such a design facilitates global feature modeling, it is limited in learning local information from HSIs. To address this limitation, we replace the FC layers with PW Conv and insert two parallel depthwise separable convolutions with kernel sizes of 3 × 3 and 5 × 5 in between, as illustrated in Figure 4.

This process can be formulated as follows:

(15) $$X_{1}={\mathrm{dwConv}}_{3\times 3}\left(\mathrm{PWConv}\left(X_{\mathrm{MMSA}}\right)\right)$$

(16) $$X_{2}={\mathrm{dwConv}}_{3\times 3}\left(\mathrm{PWConv}\left(X_{\mathrm{MMSA}}\right)\right)$$

(17) $$X_{E-\mathrm{FFN}}=\mathrm{PWConv}\left(X_{1}+X_{2}\right)$$

MMSA and E-FFN are the core submodules shared by both SMT and CMT. Their architecture is described in the SMT subsection for clarity, as CMT employs the same design.

#### 3.2.2. CMT Module

**Figure 5:**
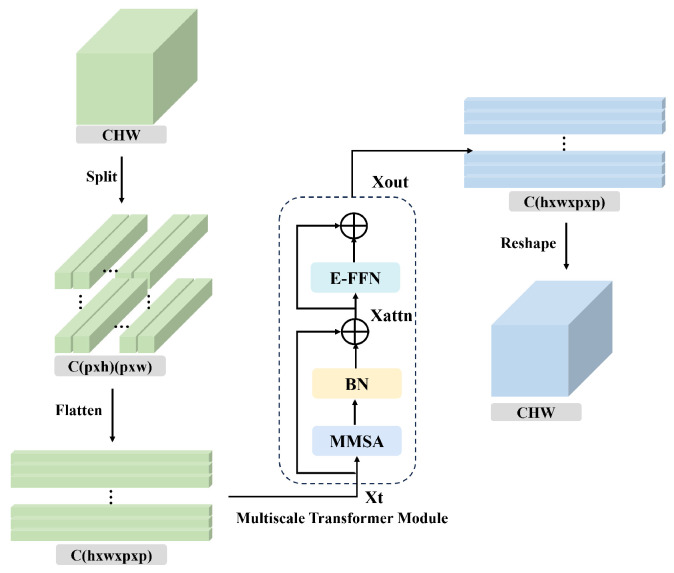
The framework of CMT.

In HU tasks, inter-channel relationships also play a critical role in enhancing unmixing performance. To further explore the channel characteristics of HSIs, we design the CMT module. In this module, the number of tokens input into the MT module is changed from the number of patches to the number of channels. The basic workflow is illustrated in Figure 5. The CMT module flattens the *N* patches into *C* tokens, where each channel is treated as an individual token and then fed into the MT module. The structure of CMT is similar to that of SMT, with the main difference being that the transformer shifts its modeling target from spatial relationships among image patches to spectral relationships among channels. By globally modeling the spectral features across different channels, the MT module effectively enhances the inter-channel spectral feature correlations within the HSI.

Therefore, SCMT sequentially processes the input through the SMT and CMT modules to capture both spatial features and spectral features across different channels, thereby providing more comprehensive and accurate representations for the HU task.

### 3.3. Unmixing with Decoder

The decoder first applies a convolutional operation to the features extracted by the encoder to generate the abundance cube $M\in R^{R\times H\times W}$. A subsequent 3 × 3 convolution is used for fine-grained refinement. To satisfy the ANC and ASC, a softmax activation function is applied along the channel dimension to obtain the estimated abundance map. To estimate the endmember signatures, the abundance matrix M is fed into the decoder branch of the AE, which consists of a single convolutional layer. This convolution expands the spectral dimension of M from *R* to *L*, producing the reconstructed HSI $\hat{X}$. The weights of this convolutional layer are initialized using endmember signatures extracted by the VCA method and are updated during training through back propagation. VCA is a widely adopted geometric-based endmember extraction technique that is known for its simplicity, efficiency, and ease of implementation, making it suitable for a broad range of HU scenarios, ultimately yielding the estimated endmember matrix $\hat{E}\in R^{L\times R}$.

### 3.4. Loss Function

Two types of loss functions are introduced during the training process of the proposed model: the reconstruction error (RE) loss and the spectral angle distance (SAD) loss. The specific formulations are given as follows:

(18) $$L_{\mathrm{RE}}\left(X,\hat{X}\right)=\frac{1}{HW}\sum_{i=1}^{H}\sum_{j=1}^{W}{\left({\hat{X}}_{ij}-X_{ij}\right)}^{2}$$

(19) $$L_{\mathrm{SAD}}\left(X,\hat{X}\right)=\frac{1}{R}\sum_{i=1}^{R}\arccos\left(\frac{\langle X_{i},{\hat{X}}_{i}\rangle }{\|X_{i}{\|}_{2}{\left\|{\hat{X}}_{i}\right\|}_{2}}\right)$$

The RE loss is computed using the mean squared error (MSE), which guides the encoder to extract essential features from the input HSIs while reducing the influence of redundant information. The SAD loss, on the other hand, is scale-invariant and helps mitigate the limitations of MSE in distinguishing endmember components caused by absolute magnitude differences. In HU tasks, the combination of these two loss functions not only compensates for their individual shortcomings but also accelerates the convergence of the model.

The total loss function is defined as a weighted sum of these two loss terms:

(20) $$L=\beta L_{\mathrm{RE}}+\gamma L_{\mathrm{SAD}}$$

where β and γ are regularization parameters used to balance the contributions of the two loss terms.

## 4. Experiments

### 4.1. Datasets

**Figure 6:**
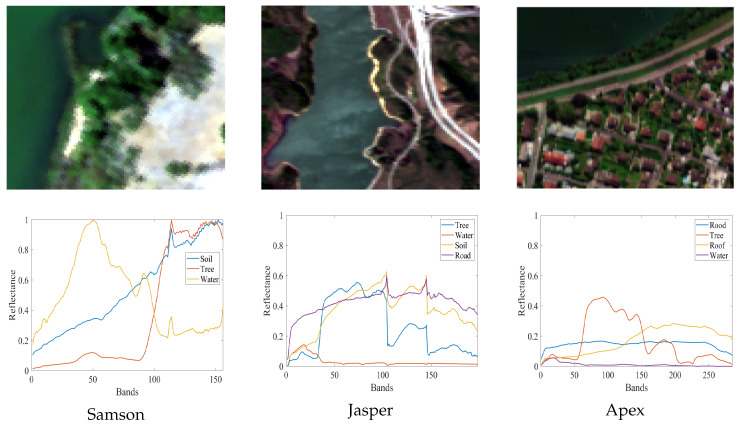
True color images and reference endmembers of the experimental datasets.

In this study, three real HSIs and one synthetic dataset were used to evaluate the performance of the proposed algorithm. Figure 6 shows the true color images and the corresponding reference endmembers of the real datasets.

(1) Samson Dataset [45]: Collected by the SAMSON sensor, this dataset consists of 952 × 952 pixels with 156 spectral bands ranging from 401 to 889 nm. A cropped subimage of size 95 × 95 pixels was used in the experiments. The dataset contains three endmember classes: soil, tree, and water.

(2) Jasper Ridge Dataset [46]: Acquired by the AVIRIS sensor, the original image has a spatial resolution of 512 × 614 pixels and contains 224 spectral bands covering the wavelength range of 380 to 2500 nm. A 100 × 100 pixel subimage was used in the experiments. After removing bands affected by water absorption and atmospheric interference, 198 valid bands were retained. The image includes four endmember classes: soil, water, tree, and road.

(3) Apex Dataset [37]: The Apex image is acquired by the APEX sensor, consisting of 110 × 110 pixels with 285 spectral bands covering the wavelength range of 413–2421 nm. This dataset includes four endmember classes: water, tree, road, and roof.

(4) Synthetic Dataset [47]: The dataset is constructed using endmembers extracted from a real HSI and contains 50 × 50 pixels with 162 spectral bands, covering five categories of endmembers: roof, metal, soil, tree, and asphalt. It is generated based on the GLMM, which extends the LMM by introducing pixel-wise scaling factors to simulate spectral variability from terrain, illumination, or atmospheric effects. While more flexible, the mixing remains linear. This dataset helps assess the proposed method’s robustness and generalization under challenging conditions, offering a more rigorous benchmark for real-world scenarios.

### 4.2. Description of Experimental Equipment and Parameters

The experiments in this study were conducted on a PC equipped with an AMD Ryzen 7 7735H processor with Radeon Graphics (AMD, Santa Clara, CA, USA) and an NVIDIA GeForce RTX 4060 Laptop GPU (NVIDIA, Santa Clara, CA, USA) using the Python 3.8.0 interpreter. Several hyperparameters were explored across different datasets, as summarized in Table 1. The regularization parameters β and γ are employed to balance the contributions of the RE and SAD loss terms. Other parameters, including patch size P, the resolution of the feature $X_{\mathrm{Ada}}$ denoted as A, the number of training epochs, the learning rate, and the weight decay coefficient, are also listed in the table.

### 4.3. Comparison Methods

To comprehensively evaluate the effectiveness of the proposed SCMT-Net, seven representative unmixing methods were selected for comparison. Specifically, VCA [16] and FCLSU [17] were chosen as representatives of geometry-based and least-squares-based unmixing approaches, respectively. MLNMF [23] was included as a typical statistical modeling method, and the uDAS [30] was considered as an unsupervised learning-based method. In addition, six state-of-the-art DL-based unmixing models were evaluated: CyCU-Net [34], Deep-Trans [38], HUMSCAN [35], CDCTA [36], MAT-Net [39], and CMSDAE [40]. Among them, CyCU-Net focuses on spatial feature extraction, Deep-Trans is the first unmixing network based on the transformer architecture, HUMSCAN emphasizes multiscale spatial feature modeling, CDCTA addresses endmember variability while preserving spectral geometry, MAT-Net integrates CNN-extracted spectral and spatial features using a transformer-based encoder, and CMSDAE enhances channel-wise multiscale representation through spectral-channel attention mechanisms. For all comparison methods, the initial endmembers were extracted using the VCA algorithm.

### 4.4. Evaluation Metrics

The quantitative results are reported using the root mean squared error (RMSE) between the estimated abundances and the ground-truth abundances, which is calculated as follows:

(21) $$\mathrm{RMSE}\left(M,\hat{M}\right)=\sqrt{\frac{1}{RHW}\sum_{k=1}^{R}\sum_{i=1}^{H}\sum_{j=1}^{W}{\left({\hat{M}}_{kij}-M_{kij}\right)}^{2}}$$

as well as the SAD between the estimated and ground-truth endmembers, which is computed as follows:

(22) $$\mathrm{SAD}\left(S,\hat{S}\right)=\frac{1}{R}\sum_{i=1}^{R}\arccos\left(\frac{\langle S_{\left(i\right)},{\hat{S}}_{\left(i\right)}\rangle }{\|S_{i}{\|}_{2}\cdot {\left\|{\hat{S}}_{i}\right\|}_{2}}\right)$$

where “·” denotes the inner product between vectors, and $S_{\left(i\right)}$ represents the *i*-th column of the ground-truth endmember matrix **S**.

### 4.5. Quantitative Results

(1) Samson Dataset: The quantitative results on the Samson dataset are presented in Table 2 and Table 3. It can be observed that SCMT-Net significantly outperforms other methods in both abundance estimation and endmember extraction. The proposed method achieves an average RMSE of 0.0854, which is notably lower than that of the second-best method, CMSDAE. Moreover, the average SAD of SCMT-Net is 0.0389. Although CyCU-Net demonstrates the best performance for tree endmember extraction, SCMT-Net achieves the highest overall endmember estimation accuracy. These results demonstrate the strong competitiveness of SCMT-Net on the Samson dataset and further validate its feasibility and superiority in HU tasks.

(2) Jasper Dataset: The quantitative evaluation results on the Jasper Ridge dataset are presented in Table 4 and Table 5. As shown in the tables, SCMT-Net achieves an average RMSE of 0.0885, which is 19.3% lower than the second-best method, CMSDAE. The proposed method outperforms most existing techniques in abundance estimation for all four endmembers in the Jasper dataset, delivering competitive results. Although methods such as Trans-Net and CDCTA exhibit certain strengths, SCMT-Net demonstrates the most accurate estimation of endmember spectra overall, highlighting its robustness and effectiveness in HU.

(3) Apex Dataset: The quantitative evaluation results on the Apex dataset are reported in Table 6 and Table 7. SCMT-Net stands out by achieving the lowest average RMSE and SAD values, with an average RMSE of 0.1185 and an average SAD of 0.0771, indicating superior performance in both abundance and endmember estimation. The endmember “Road” in the Apex dataset poses a considerable challenge for most comparison methods, whereas SCMT-Net is capable of producing a satisfactory estimation. The Apex dataset contains richer spectral information with a greater number of spectral bands and more complex spatial features, making the unmixing task more challenging.

(4) Synthetic Dataset: The quantitative results on the synthetic dataset are presented in Table 8 and Table 9. The proposed method achieves superior performance in both abundance estimation and endmember signature reconstruction, with improvements of 15.4% and 30%, respectively, over the second-best methods. Even under non-ideal conditions with pixel-level endmember variability, the proposed SCMT-Net consistently maintains the lowest average RMSE and SAD values, demonstrating its robustness and practical applicability in complex mixing scenarios.

### 4.6. Visual Analysis

**Figure 7:**
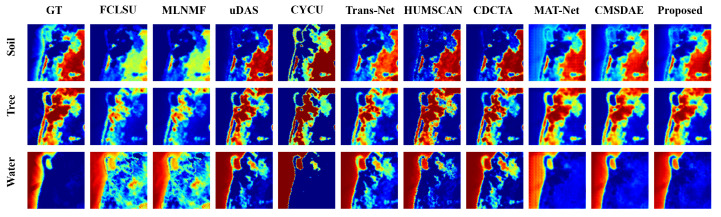
Visual comparison of abundance maps obtained by different unmixing methods on the Samson dataset.

**Figure 8:**
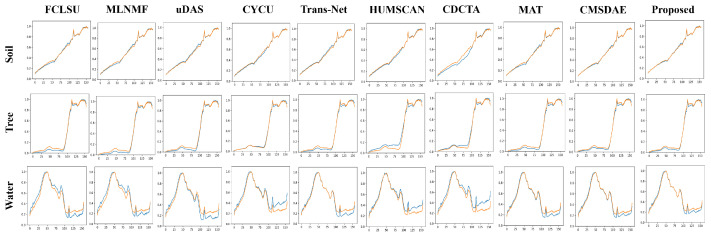
Visual comparison of endmembers obtained by different unmixing methods on the Samson dataset. Ground-truth endmembers are shown in blue, and estimated endmembers are shown in orange.

For the Samson dataset, the abundance estimation results are illustrated in Figure 7. The proposed SCMT-Net produces abundance maps that are highly consistent with the ground truth, which can be attributed to its effective utilization of multiscale spatial and channel information, enabling precise capture of fine-grained features in fragmented regions. In contrast, methods such as FCLSU and MLNMF tend to underestimate the abundance of soil and tree, resulting in over-detailed water abundance maps. Moreover, Trans-Net and CDCTA overestimate the abundance of water in some tree-covered areas. The abundance maps generated by CyCU-Net lack the detail and smoothness observed in the reference maps. Figure 8 shows the endmember estimation results, where the endmembers extracted by the proposed method are almost identical to the ground-truth signatures.

**Figure 9:**
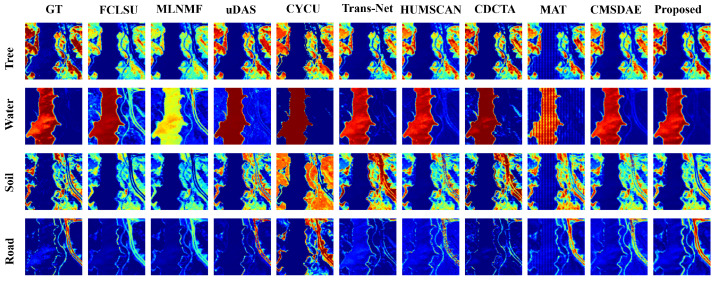
Visual comparison of abundance maps obtained by different unmixing methods on the Jasper dataset.

**Figure 10:**
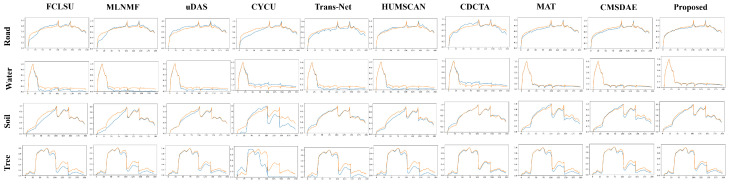
Visual comparison of endmembers obtained by different unmixing methods on the Jasper dataset. Ground-truth endmembers are shown in blue, and estimated endmembers are shown in orange.

For the Jasper dataset, Figure 9 and Figure 10 present a visual comparison of unmixing results obtained by different methods. The abundance maps and endmember spectra generated by SCMT-Net are more consistent with the reference data. Methods such as FCLSU and MLNMF tend to overestimate the abundance of water while underestimating those of trees and roads, and the soil abundance maps they produce lack fine details. SCMT-Net demonstrates particularly strong performance in estimating the “tree” endmember. In the Jasper dataset, roads occupy a small portion of the scene, making the estimation of their abundance and spectral signatures more challenging due to complex distributions. Many methods fail to accurately estimate the abundance of roads or to fully separate them, whereas SCMT-Net achieves more precise separation. These results further confirm that the proposed network is more effective in capturing fine-grained details and contextual information in HSIs compared to other DL approaches.

**Figure 11:**
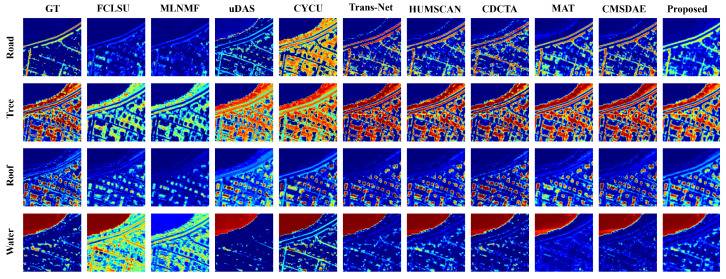
Visual comparison of abundance maps obtained by different unmixing techniques on the Apex dataset.

**Figure 12:**
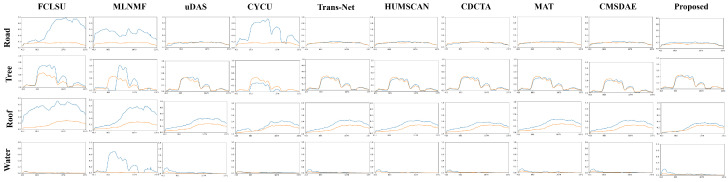
Visual comparison of endmembers obtained by different unmixing methods on the Apex dataset. Ground-truth endmembers are shown in blue, and estimated endmembers are shown in orange.

For the Apex dataset, the abundance maps shown in Figure 11 indicate that the results produced by SCMT-Net are visually closest to the reference. FCLSU and MLNMF significantly overestimate the abundance of water and fail to provide accurate estimation for roads. The water abundance map generated by SCMT-Net avoids the redundant textures and erroneous details commonly observed in other methods, demonstrating higher accuracy, particularly in complex mixed-pixel regions. Compared with other approaches, SCMT-Net effectively suppresses unnecessary estimation errors and ensures proper separation between water and other materials. As shown in Figure 12, FCLSU, MLNMF, and CyCU-Net fail to correctly extract the road endmember. Although HUMSCAN achieves the best performance for the “Water” endmember category, the proposed method shows superior overall performance in endmember estimation.

**Figure 13:**
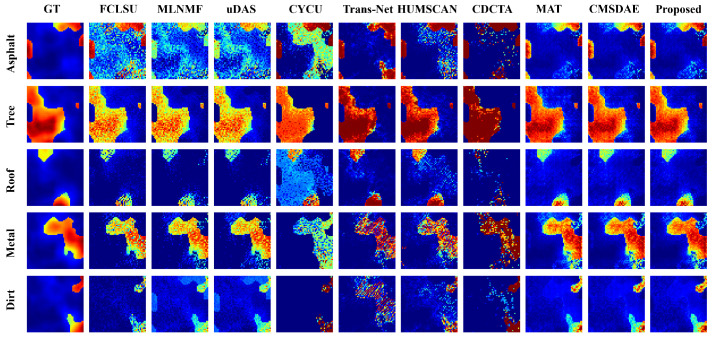
Visual comparison of abundance maps obtained by different unmixing techniques on the synthetic dataset.

**Figure 14:**
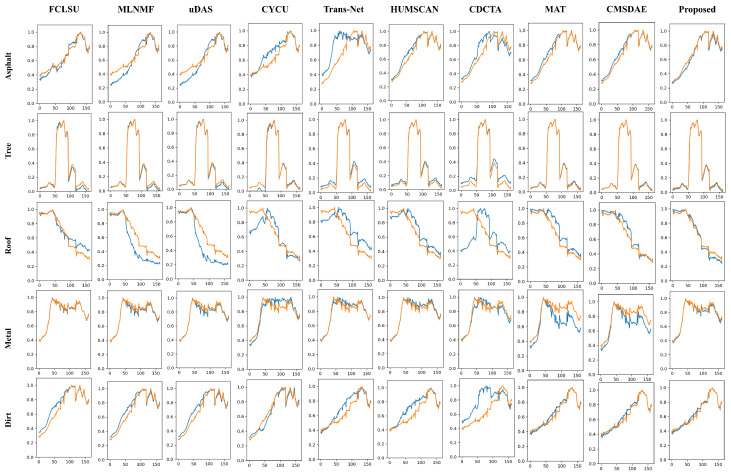
Visual comparison of endmembers obtained by different unmixing methods on the synthetic dataset. Ground-truth endmembers are shown in blue, and estimated endmembers are shown in orange.

For the synthetic dataset, as shown in the visual results of Figure 13, although most methods can roughly distinguish different materials in the scene, SCMT-Net achieves the reconstruction results closest to the reference. As illustrated in Figure 14, CDCTA shows a significant deviation in estimating the “Roof” endmember, while FCLSU achieves the best performance on the “Metal” endmember. Nevertheless, the proposed method demonstrates superior overall performance in endmember estimation. It is worth noting that even under non-ideal conditions where pixel-level endmember variability is present—conditions that deviate from the assumptions of the LMM—SCMT-Net is still able to maintain stable and accurate unmixing performance. These results indicate its strong generalization capability and robustness, making it well suited for HU tasks in complex mixing scenarios.

### 4.7. Ablation Study

We adopted an AE architecture to perform unmixing tasks on four different datasets. To validate the contribution of each encoder module in SCMT-Net, ablation experiments were conducted accordingly.

(1) Effect of Each Module of SCMT-Net: Table 10 presents the ablation results of different module combinations in the SCMT encoder across multiple datasets. Compared with the full configuration where all three modules work cooperatively, the unmixing performance degrades when any individual module is used alone. This indicates that each component plays a complementary role. As transformer architectures are particularly effective at modeling long-range dependencies, the inclusion of both the SMT and CMT modules significantly enhances the unmixing capability of the SCMT encoder.

(2) Effect of MMSA Module: Table 11 investigates the performance contributions of the multiscale branch and the channel attention branch within the MMSA module. When only the multiscale branch is used, the lack of channel-wise information interaction leads to performance degradation, indicating that the channel attention branch in MMSA is critical—while introducing negligible computational overhead. Conversely, when only the channel attention branch is used, the absence of multiscale contextual information also results in suboptimal performance. These results demonstrate that using either component alone is less effective than combining both, highlighting the complementary nature of multiscale and channel attention mechanisms in HU.

### 4.8. Computational Complexity and Time Consumption Analysis

To compare the model complexity and computational efficiency of different methods, all experiments were conducted on the same computing platform. Model complexity was evaluated by calculating the number of parameters and floating-point operations (FLOPs), while computational efficiency was assessed based on runtime. All experiments were performed on the Jasper dataset, and the results are presented in Table 12.

Traditional methods exhibit high computational efficiency with a relatively low processing time. Among the deep learning-based approaches, CDCTA and HUMSCAN show longer runtimes due to their complex network architectures, whereas the proposed SCMT-Net demonstrates more competitive computational efficiency. Among all the compared methods, SCMT-Net achieves the best unmixing accuracy while maintaining relatively low parameter counts and FLOPs, along with a shorter runtime. Since model complexity remains an important consideration, we will further optimize the architecture of SCMT-Net in our future work.

## 5. Conclusions

This paper proposes a novel HU network, SCMT-Net, which combines the CFP module and a hybrid model of the SCMT module to achieve the collaborative modeling of local details and a global context. In particular, the CMT module we designed excels at capturing long-range dependencies between channels, while the MMSA module embedded in both the SMT and CMT promotes the representation of multiscale features. Coupled with the E-FFN, it further enhances the information interaction between different channels, effectively learning the dynamic relationships between multiscale spatial and spectral features. Through comparative analysis on multiple datasets, SCMT-Net demonstrated superior performance in abundance estimation and endmember extraction tasks, validating its strong generalization ability and outstanding feature representation capabilities. Although SCMT-Net demonstrated strong performance on multiple HU datasets, it still has certain limitations. The proposed model has room for improvement in terms of parameter size and computational overhead, and future work will focus on designing more lightweight network architectures while maintaining unmixing performance. In addition, the channel feature modeling process may be affected by redundant information, potentially weakening the representation of high-dimensional spectral features. Future research will consider introducing more efficient attention mechanisms to optimize the channel modeling strategy and incorporating feature aggregation enhancement modules to further improve the model’s accuracy, robustness, and practicality.

## Figures and Tables

**Table 1:** Hyperparameter settings.

Hyperparameters	Synthetic	Samson	Jasper	Apex
P	(5 × 5)	(5 × 5)	(5 × 5)	(5 × 5)
A	10	9	10	8
β	8 × 10^2^	9 × 10^2^	2 × 10^3^	5 × 10^2^
γ	9 × $10^{-2}$	11 × $10^{-2}$	5 × $10^{-2}$	5 × $10^{-2}$
Epoch	250	220	140	250
Learning rate	8 × $10^{-3}$	6 × $10^{-3}$	4 × $10^{-3}$	9 × $10^{-2}$
Weight decay	6 × $10^{-5}$	5 × $10^{-4}$	22 × $10^{-5}$	4 × $10^{-4}$

**Table 2:** Average RMSE results on the Samson dataset.

Class	FCLSU	MLNMF	uDAS	CYCU	Trans-Net	HUMSCAN	CDCTA	MAT	CMSDAE	Proposed
Soil	0.2420	0.2396	0.1560	0.1780	0.1608	0.1624	0.1773	0.1115	0.1017	**0.0925**
Tree	0.2385	0.2390	0.1168	0.1652	0.1520	0.1043	0.1595	0.0560	**0.0653**	0.0581
Water	0.3851	0.3861	0.2355	0.1648	0.2661	0.1872	0.2866	0.1042	0.1030	**0.0997**
**Mean RMSE**	0.2965	0.2964	0.1765	0.1695	0.1998	0.1552	0.2152	0.0939	0.0917	**0.0854**

**Table 3:** Average SAD results on the Samson dataset.

Class	VCA	MLNMF	uDAS	CYCU	Trans-Net	HUMSCAN	CDCTA	MAT	CMSDAE	Proposed
Soil	0.0248	0.0248	0.0207	0.0210	0.0186	0.0237	0.0691	0.0221	0.0137	**0.0134**
Tree	0.0518	0.0528	0.0492	**0.0282**	0.0465	0.0871	0.0487	0.0471	**0.0410**	0.0420
Water	0.1093	0.0983	0.1299	0.1101	0.0902	0.0859	0.1232	0.0741	0.0632	**0.0613**
**Mean SAD**	0.0620	0.0586	0.0666	0.0531	0.0518	0.0656	0.0803	0.0478	0.0393	**0.0389**

**Table 4:** Average RMSE results on the Jasper dataset.

Class	FCLSU	MLNMF	uDAS	CYCU	Trans-Net	HUMSACN	CDCTA	MAT	CMSDAE	Proposed
Tree	0.1558	0.1547	0.1267	0.1887	0.1336	0.1158	0.0820	0.1444	0.1258	**0.0786**
Water	0.1965	0.3422	0.1509	0.0991	0.0557	0.0634	0.0919	0.0992	0.0521	**0.0460**
Soil	0.1393	0.1397	0.1194	0.2946	0.2324	0.1316	0.1978	0.1276	0.1225	**0.1178**
Road	0.1087	0.1037	0.1184	0.2686	0.1953	0.1391	0.1760	**0.0936**	0.1213	0.0956
**Mean RMSE**	0.1534	0.2069	0.1295	0.2260	0.1681	0.1163	0.1460	0.1180	0.1098	**0.0885**

**Table 5:** Average SAD results on the Jasper dataset.

Class	VCA	MLNMF	uDAS	CYCU	Trans-Net	HUMSCAN	CDCTA	MAT	CMSDAE	Proposed
Road	0.0901	0.1017	0.0361	0.1023	0.0606	0.0373	0.0370	0.0458	0.0417	**0.0304**
Water	0.2554	0.2929	0.4101	0.1770	0.2574	0.2567	0.2368	0.0609	0.0461	**0.0426**
Soil	0.1166	0.1630	0.0532	0.2839	**0.0465**	0.0652	**0.0449**	0.1123	0.0879	0.0531
Tree	0.1657	0.1337	0.1509	0.5241	0.1834	0.1925	0.1041	0.1247	0.0991	**0.0752**
**Mean SAD**	0.1569	0.1728	0.1626	0.2718	0.1370	0.1379	0.1057	0.0859	0.0687	**0.0503**

**Table 6:** Average RMSE results on the Apex dataset.

Class	FCLSU	MLNMF	uDAS	CYCU	Trans-Net	HUMSCAN	CDCTA	MAT	CMSDAE	Proposed
Road	0.1827	0.1686	0.2578	0.3400	0.1946	0.1630	0.2171	0.1568	0.1786	**0.1379**
Tree	0.2427	0.2388	0.4781	0.2550	0.1215	0.1011	0.1262	0.1262	0.1263	**0.1236**
Roof	0.2101	0.2015	0.3352	0.1213	0.1375	0.1185	0.1206	0.1433	0.1269	**0.1148**
Water	0.3827	0.7081	0.3845	0.1278	0.1146	0.0937	0.1027	0.1371	0.1367	**0.0931**
**Mean RMSE**	0.2660	0.3961	0.3726	0.2300	0.1455	0.1221	0.1485	0.1413	0.1437	**0.1185**

**Table 7:** Average SAD results on the Apex dataset.

Class	VCA	MLNMF	uDAS	CYCU	Trans-Net	HUMSCAN	CDCTA	MAT	CMSDAE	Proposed
Road	0.4775	0.1402	0.1485	0.4312	0.1081	0.1194	0.1454	0.1245	0.1260	**0.0614**
Tree	0.1287	0.8179	0.1350	0.2476	0.1353	0.1403	0.1347	0.1369	0.1321	**0.1319**
Roof	0.3660	**0.0709**	0.1078	0.1102	0.1024	0.1143	0.2000	0.1145	0.1150	**0.0756**
Water	0.2123	1.1913	0.0616	0.4204	0.0397	**0.0388**	0.0432	0.0432	0.0415	**0.0395**
**Mean SAD**	0.2961	0.5551	0.1132	0.3024	0.0964	0.1032	0.1308	0.1048	0.1036	**0.0771**

**Table 8:** Average RMSE results on the Synthetic dataset.

Class	FCLSU	MLNMF	uDAS	CYCU	Trans-Net	HUMSCAN	CDCTA	MAT	CMSDAE	Proposed
Asphalt	0.2818	0.3158	0.2813	0.3033	0.2614	0.1874	0.2318	0.0690	0.0685	**0.0686**
Tree	0.1111	0.0736	0.0809	0.0750	0.0952	0.0702	0.1223	0.0540	0.0483	**0.0400**
Roof	0.1190	0.1762	0.1335	0.1452	0.0777	0.0824	0.1991	0.0803	0.0729	**0.0613**
Metal	0.1175	0.1395	0.1104	0.2853	0.2132	0.1933	0.2142	0.1096	0.1008	**0.0782**
Dirt	0.1292	0.1670	0.1333	0.1690	0.3169	0.1215	0.1304	0.0835	0.0823	**0.0687**
**Mean RMSE**	0.1652	0.1916	0.1633	0.2138	0.2142	0.1407	0.1851	0.0814	0.0765	**0.0647**

**Table 9:** Average SAD results on the Synthetic dataset.

Class	FCLSU	MLNMF	uDAS	CYCU	Trans-Net	HUMSCAN	CDCTA	MAT	CMSDAE	Proposed
Asphalt	0.0586	0.1329	0.1324	0.0969	0.2103	0.0527	0.1277	0.0474	0.0431	**0.0338**
Tree	0.0660	0.0453	0.0465	0.0908	0.0845	0.0490	0.1248	**0.0211**	**0.0132**	0.0271
Roof	0.1003	0.1887	0.2339	0.1823	0.1835	0.1088	0.4323	0.0851	0.0643	**0.0570**
Metal	**0.0080**	0.0178	0.0217	0.0746	0.0350	0.0179	0.0633	0.1029	0.0716	**0.0153**
Dirt	0.0760	0.0647	0.0681	0.0596	0.0999	0.1043	0.2156	**0.0435**	0.0440	**0.0318**
**Mean SAD**	0.0618	0.0899	0.1005	0.1008	0.1226	0.0665	0.1927	0.0600	0.0472	**0.0330**

**Table 10:** Ablation study of different encoder module combinations on the four datasets.

Dataset	CFP		CFP + SMT		CFP + CMT		Proposed	
	RMSE	SAD	RMSE	SAD	RMSE	SAD	RMSE	SAD
Samson	0.1242	0.0729	0.1149	0.0590	0.0958	0.0678	**0.0854**	**0.0389**
Jasper	0.1313	0.0822	0.1144	0.0731	0.1459	0.0774	**0.0885**	**0.0503**
Apex	0.1876	0.1409	0.1901	0.1629	0.1872	0.1214	**0.1185**	**0.0771**
Synthetic	0.0835	0.0348	0.1103	0.0592	0.1034	0.0337	**0.0647**	**0.0330**

**Table 11:** Ablation study of the MMSA module on the four datasets.

Module	Multiscale	Channel	Samson		Jasper		Apex		Synthetic	
			RMSE	SAD	RMSE	SAD	RMSE	SAD	RMSE	SAD
MMSA	✓		0.0918	0.1070	0.1329	0.0738	0.1455	0.0964	0.1265	0.1409
MMSA		✓	0.1123	0.0905	0.1296	0.0774	0.1245	0.1175	0.1396	0.1649
MMSA	✓	✓	**0.0854**	**0.0389**	**0.0885**	**0.0503**	**0.1185**	**0.0771**	**0.0647**	**0.0330**

**Table 12:** Computational complexity and time consumption comparison on the Jasper dataset.

Method	FCLSU	MLNMF	uDAS	CYCU	Trans-Net	HUMSACN	CDCTA	MAT	CMSDAE	Proposed
aRMSE	0.1534	0.2069	0.1295	0.2260	0.1681	0.1163	0.1460	0.1180	0.1098	**0.0885**
aSAD	0.1569	0.1728	0.1626	0.2718	0.1370	0.1379	0.1057	0.0859	0.0687	**0.0503**
Params	-	**0.005 M**	0.007 M	0.29 M	7.75 M	9.64 M	8.37 M	6.79 M	6.6 M	7.69 M
FLOPs	-	71.23 K	**17.52 K**	0.35 M	2.97 G	4.89 G	5.23 G	2.89 G	5.13 G	4.76 G
Computational Time	0.69 s	2.36 s	6.56 s	7.12 s	22.35 s	67.21 s	72.69 s	23.69 s	24.72 s	**23.85 s**

## Data Availability

Some datasets are available at https://rslab.ut.ac.ir/data (accessed on 14 May 2025).
